# Basic and clinical immunology – 3023. Influence of fexofenadine hydrochloride on uteroglobin production from nasal epithelial cells *in vitro and in vivo*

**DOI:** 10.1186/1939-4551-6-S1-P199

**Published:** 2013-04-23

**Authors:** Kenichi Kanai, Kazuhito Asano, Atsuko Furuta, Takeyuki Sanbe, Harumi Suzaki

**Affiliations:** 1 Otorhinolaryngology, Showa University, Tokyo, Japan; 2 Physiology, Showa University, Japan

## Background

Uteroglobin (CC10) is well known to be an immuno-suppressive protein secreted from airway epithelial cells after inflammatory stimulation and function in development of allergic disorders. Although histamine H_1_ receptor antagonists are used for the treatment of allergic disorders, the influence of the agents on CC10 production is not well understood. In the present study, we examined the influence of a histamine H_1_ receptor antagonist, fexofenadine hydrochloride (FEX) on CC10 production *in vitro* and *in vivo.*

## Methods

Nasal epithelial cells (5 x 10^6^ cells/ml) were stimulated with 20 ng/ml TNF-α in the presence of various concentrations of FEX for 24 h. CC10 levels in culture supernatants were examined by ELISA. Patients with Japanese cedar pollinosis were orally treated with FEX twice a day at a single dose of 60 mg for two weeks during Japanese cedar pollen season (February 2011 to April 2011). CC10 levels in nasal secretions were also examined by ELISA.

## Results

The addition of FEX into epithelial cell cultures caused dose-dependent increase in the ability of cells to produce CC10 in response to TNF-α stimulation, and the minimum concentration that caused significant increase was 200 ng/ml. Oral administration of FEX for two weeks also increased CC10 levels in nasal secretions from pollinosis patients along with attenuation of clinical symptoms.

## Conclusions

The ability of FEX to enhance CC10 production may account, at least in part, for the clinical efficacy of the agent on allergic disorders, including allergic rhinitis

